# Supplementary material for: Content-rich biological network constructed by mining PubMed abstracts
Source: BMC Bioinformatics. 2004 Oct 8;5:147. doi: 10.1186/1471-2105-5-147 (PMC528731; doi:10.1186/1471-2105-5-147)
Supplement: Additional File 2 — The original results of the above study (non-essential files are deleted to keep the file size under the limit set by BMC bioinformatics). [file 1471-2105-5-147-S2.bz2 › chilibotAdditionalFile2/dip10/203ID7664335E693/html/ERCC6_CKN1.html]

 


 **ERCC6** and **CKN1** 
  
Found 57 abstracts in PubMed, retrieved 10.  
 

 What does Google say? 
 PDF only 
| .edu only 

---

**Interactive relationship** (e.g. stimulation, inhibition, etc)

**Neutral relationship**- The hydrogen peroxide induced translocation of CSA  [ **CKN1** ]  was also CSB  [ **ERCC6** ]  dependent.  Ref: 11782547 Proc Natl Acad Sci U S A, 2002
- The translocation of CSA  [ **CKN1** ]  was independent of Xeroderma pigmentosum group C, which is specific to the global genome repair subpathway of nucleotide excision repair and of the core NER factor Xeroderma pigmentosum group A but required the CSB  [ **ERCC6** ]  protein.  Ref: 11782547 Proc Natl Acad Sci U S A, 2002

**Non-interactive relationship** (e.g. studied together, co-existance, homology, etc.)

- Cockayne syndrome CS is a segmental premature aging syndrome in humans that has two complementation groups, CSA  [ **CKN1** ]  and CSB  [ **ERCC6** ] .  Ref: 12483520 Oncogene, 2002
- Two genes are involved CSA  [ **CKN1** ]  and CSB  [ **ERCC6** ] .  Ref: 12560492 Nucleic Acids Res, 2003
- Despite this DNA repair defect, individuals with CS of which there are two complementation groups, CSA  [ **CKN1** ]  and CSB  [ **ERCC6** ]  do not demonstrate an elevated incidence of cancer.  Ref: 11713576 Int J Oncol, 2001
- Traditionally, CSA  [ **CKN1** ]  and CSB  [ **ERCC6** ]  have been ascribed a function in the transcription coupled repair pathway of nucleotide excision repair that efficiently removes bulky lesions from the transcribed strand of RNA polymerase II transcribed genes.  Ref: 12032859 Oncogene, 2002
- The chondroitin sulfates CSA  [ **CKN1** ] , CSB  [ **ERCC6** ] , CSC had far less impact upon APTT with the FXa FXDP system.  Ref: 12430983 Can J Physiol Pharmacol, 2002
- The disease is caused by the disruption of CSA  [ **CKN1** ] , CSB  [ **ERCC6** ] , or some types of xeroderma pigmentosum genes.  Ref: 12665480 FASEB J, 2003
- ... survival, repair and recovery from transcription inhibition critically depend on CSA  [ **CKN1** ] , CSB  [ **ERCC6** ]  and UVS, the factors specific for transcription coupled NER.  Ref: 12531012 DNA Repair Amst , 2002
- Comparison of the activities of CSA  [ **CKN1** ]  and CSB  [ **ERCC6** ]  oligo and polysaccharides with a similar sulfation pattern and content achieved from partial desulfation demonstrated that glucuronic acid rather than iduronic acid residues are important for IRBC binding.  Ref: 11956186 J Biol Chem, 2002
